# Supplementary figures and images for: Co-applied biochar and drought tolerant PGPRs induced more improvement in soil quality and wheat production than their individual applications under drought conditions
Source: PeerJ. 2024 Oct 25;12:e18171. doi: 10.7717/peerj.18171 (PMC11514766; doi:10.7717/peerj.18171)

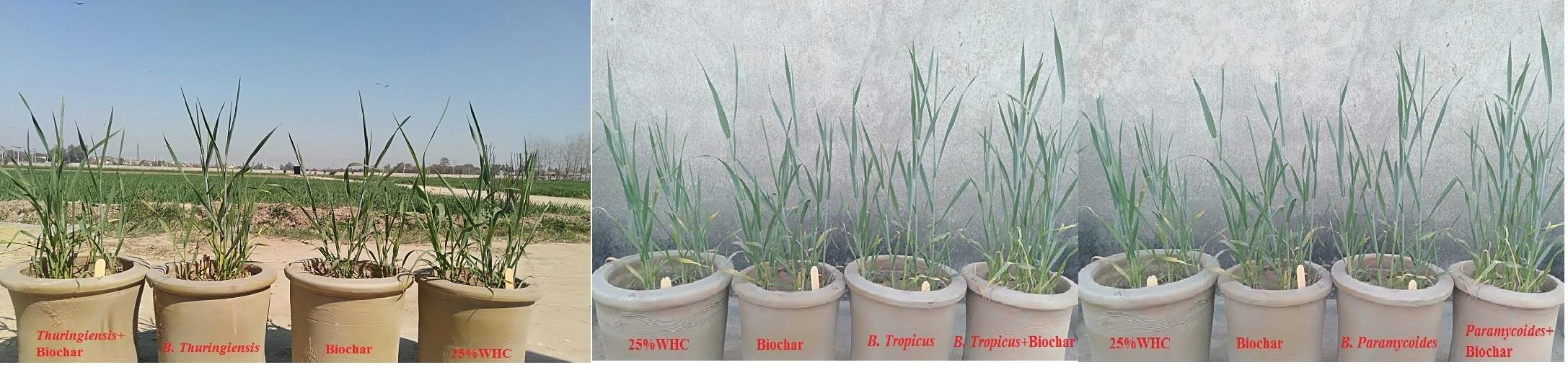

Supplement: Supplemental Information 1 [file peerj-12-18171-s001.png]
